# Metabolic dysfunction-associated fatty liver disease increased the risk of subclinical carotid atherosclerosis in China

**DOI:** 10.3389/fendo.2023.1109673

**Published:** 2023-04-04

**Authors:** Fang Lei, Xiao-Ming Wang, Changquan Wang, Xuewei Huang, Ye-Mao Liu, Juan-Juan Qin, Peng Zhang, Yan-Xiao Ji, Zhi-Gang She, Jingjing Cai, Huo-ping Li, Xiao-Jing Zhang, Hongliang Li

**Affiliations:** ^1^ Department of Cardiology, Renmin Hospital, School of Basic Medical Science, Wuhan University, Wuhan, China; ^2^ Institute of Model Animal, Wuhan University, Wuhan, China; ^3^ Department of Neurology, Huanggang Central Hospital of Yangtze University, Huanggang, China; ^4^ Huanggang Institute of Translational Medicine, Huanggang Central Hospital of Yangtze University, Huanggang, China; ^5^ Department of Cardiology, The Third Xiangya Hospital, Central South University, Changsha, China; ^6^ Department of Cardiology, Huanggang Central Hospital of Yangtze University, Huanggang, China; ^7^ Medical Science Research Center, Zhongnan Hospital of Wuhan University, Wuhan, China

**Keywords:** MAFLD, subclinical carotid atherosclerosis, CIMT, association, hepatic steatosis index

## Abstract

**Background and aims:**

Metabolic dysfunction-associated fatty liver disease (MAFLD) was proposed to substitute NAFLD in 2020. This new term highlights the systematic metabolic disturbances that accompany fatty liver. We evaluated the correlations between MAFLD and subclinical carotid atherosclerosis (SCA) based on a nationwide health examination population in China.

**Methods:**

We performed a nationwide cross-sectional population and a Beijing retrospective cohort from 2009 to 2017. SCA was defined as elevated carotid intima-media thickness. The multivariable logistic and Cox models were used to analyze the association between MAFLD and SCA.

**Results:**

153,482 participants were included in the cross-sectional study. MAFLD was significantly associated with SCA in fully adjusted models, with an odds ratio of 1.66; 95% confidence interval (CI): 1.62-1.70. This association was consistent in the cohort, with a hazard ratio (HR) of 1.31. The association between baseline MAFLD and incident SCA increased with hepatic steatosis severity. Subgroup analysis showed an interaction between age and MAFLD, with a higher risk in younger groups (HR:1.67, 95% CI: 1.17-2.40).

**Conclusion:**

In this large cross-section and cohort study, MAFLD was significantly associated with the presence and development of SCA. Further, the risk was higher among MAFLD individuals with high hepatic steatosis index and young adults.

## Introduction

Non-alcoholic fatty liver disease (NAFLD) is a highly prevalent metabolic disorder that is tightly associated with the overweight and obesity epidemic worldwide, placing a huge burden on families, individuals, and healthcare systems. The serious complications of NAFLD are not limited to advanced cirrhosis and liver cancer but also lead to cardiovascular diseases (1–4). Several studies, including retrospective and prospective, have shown that the presence and extent of NAFLD are associated with increased prevalence and incidence of cardiovascular disease (CVD). Our previous meta-analysis demonstrated that nearly 55% of NAFLD patients have CVDs in China (5, 6).

Metabolic dysfunction-associated fatty liver disease (MAFLD) was proposed to substitute NAFLD in 2020. This new term highlights the systematic metabolic disturbances that accompany fatty liver. Our previous study based on a nationwide health examination population in China showed that the prevalence of MAFLD rose rapidly from 22.8% to 35.6% in 2009-2017 (7). Subclinical atherosclerosis was considered a precursor to clinical atherosclerosis and is significantly associated with a high risk of CVDs. Carotid intima-media thickness (CIMT) and carotid plaque were imaging indexes of subclinical carotid atherosclerosis (SCA) (8, 9). Previous studies demonstrated that NAFLD was strongly correlated with a high risk of subclinical atherosclerosis (2, 10). A meta-analysis of more than 20 cross-sectional studies revealed a strong independent association between NAFLD and subclinical atherosclerosis, demonstrated by CIMT, coronary artery calcification, endothelial dysfunction and arterial stiffness (11). Furthermore, a community-based cohort showed that during 4.3 years, MAFLD was significantly correlated with a high risk of subclinical atherosclerosis, as demonstrated by CIMT, microalbuminuria, and baPWV (12). However, evidence regarding the hazards of developing subclinical arteriosclerosis as measured by CIMT and carotid plaque with MAFLD based on a large population in China remains to be explored.

Therefore, we aimed to explore the correlations of a new definition MAFLD and SCA based on a cross-sectional study and a retrospective cohort based on nationwide health examination. Further stratification was conducted to analyze the potential effect of age, sex, and degree of hepatic steatosis on these associations.

## Materials and methods

### Study population

**Figure 1:**
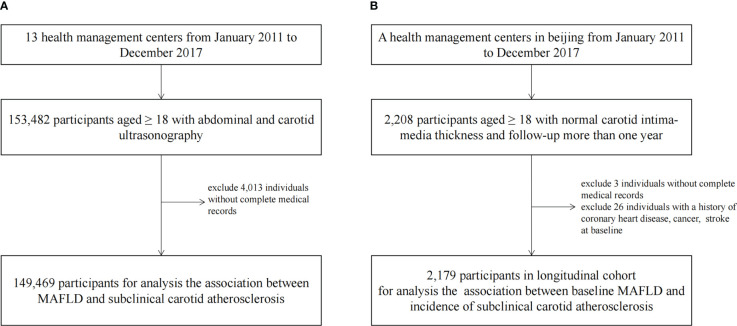
The Flowchart Showing the Strategy of Participant Enrollment. A schematic overview illustrating participants enrollment and the exclusion and inclusion criteria. **(A)** The flowchart of the cross-sectional study. **(B)** The flowchart of the cohort.

This cross-sectional study was conducted in a nationwide health examination population in China. From January 2011 to December 2017, 153,482 participants aged ≥ 18 from 13 health management centers were enrolled. We excluded 4,013 individuals without a complete medical record on MAFLD measurements. Finally, 149,469 populations were included in a cross-sectional study to explore the relationship between MAFLD and SCA (**Figure 1A**).

We conducted a retrospective cohort in the population of a Beijing health management center. The individuals had at least two carotid ultrasound examinations over a one-year follow-up between January 1, 2011 to December 31, 2017 (n=2,205). We excluded subjects without complete medical records (n=3) or a history of coronary heart disease, cancer, or stroke at baseline (n=26). The final sample size was 2,179 for analyzing the correlations of baseline MAFLD and incidence of SCA in the cohort (**Figure 1B**).

The subjects who attended the check-ups were mixed population from nearby urban and rural areas (13). The population was mainly adults consisted of population with very diverse socioeconomic and occupational backgrounds, including public services employees, doctors, workers, farmers, self-employed persons, and et al. Over 500 million people with diverse socioeconomic backgrounds (public service employees, workers, self-employed persons, farmers, and others) participate in health examinations in China each year (14, 15). The statistics showed that approximately 60% of the participants were encouraged by their employers to undergo health examinations, which were offered free of charge (15). And Participation in the health examinations was voluntary.

The study was approved by the central ethics board of Renmin Hospital of Wuhan University, followed by the acceptance with the ethics center in each collaborating hospital. Individual identification data was removed, and only anonymous information was kept during the study process. The ethics committees from each hospital waived patient informed consent.

### Data collection

All the participants underwent a detailed physical examination and completed a questionnaire that included personal and family history of disease and medication use in the health examination center. Anthropometric measurements, including height, weight, and waist circumference (WC), were performed by physicians according to standard protocols. Blood pressure (BP) was measured after subjects were quietly seated for 10 minutes. After overnight fasting, routine blood tests, biochemical blood tests, and abdominal ultrasounds were measures as part of the health check-up exam.

T2DM was defined according to the Chinese criteria and personal disease history (16). Hypertension was defined according to the national guidelines in China and personal disease history (17). Dyslipidemia was diagnosed according to the guidelines for managing dyslipidemia in adults or using the lipid-lowering drug or self-reported history of dyslipidemia (18). Hyperuricemia was diagnosed as serum uric acid ≥ 420 μmol/L in males and ≥ 360 μmol/L in females or using acid-reducing drug (19). Metabolic syndrome (MetS) was diagnosed according to the consensus criteria in 2009 (20). The hepatic steatosis index (HSI) was calculated according to the existing equation: HSI = BMI (+2, if diabetes mellitus; +2, if female) + (ALT/AST) × 8 (21). The overall HSI was then categorized into low-, middle-, and high-HSI tertiles according to the tri-sectional quantiles range. The Liver fibrosis was calculated by the fibrosis 4 score (FIB-4) (22). The estimated glomerular filtration rate (eGFR) was measured using the Modification of Diet in Renal Disease (MDRD) formula (23). Chronic kidney disease (CKD) was diagnosed as the eGFR < 60 ml/min/1.73 m^2^ (24).

### Definitions of MAFLD

MAFLD was defined by ultrasound-detected hepatic steatosis with evidence of metabolic dysregulation, overweight/obesity (BMI ≥ 23 kg/m^2^), or the presence of T2MD. Metabolic dysregulation was defined as two or more metabolic risk abnormalities: a) central obesity: WC ≥90/80 cm in males and females; b) hypertension: BP ≥130/85 mmHg or receiving specific drug treatment; c) TG ≥1.70 mmol/L or receiving specific drug treatment; d) HDL-c ≥1.0/1.3 mmol/L in males and females or receiving specific drug treatment; and e) prediabetes: FBG 5.6 to 6.9 mmol/L or 2-hour post-load glucose 7.8 to 11.0 mmol/L or glycated hemoglobin 5.7% to 6.4% (25, 26). The diagnosis of hepatic steatosis on ultrasound was based on the presence of hepatorenal echo contrast, liver parenchymal brightness, deep attenuation, and vascular blurring (26, 27).

### Definitions of subclinical carotid atherosclerosis

The examination of the cervical carotid artery was performed by B-mode ultrasonography. The specific examination sites included bilateral scans of the internal carotid arteries, external carotid arteries, common carotid arteries, and bifurcation sites. We defined elevated CIMT as a CIMT value > 1.2 mm. In any carotid segment, focal wall thickening or CIMT > 1.5 mm was identified as carotid plaque. SCA was diagnosed as having carotid plaque or increased CIMT (28).

### Statistical analysis

All statistical analyses were performed by R-3.6.3 (R Foundation for Statistical Computing, Vienna, Austria). Continuous variables were reported as means ± standard deviations (sd) or medians and inter-quartile ranges (IQRs). Categorical variables were reported in absolute numbers (percentages). ANOVA was used for comparisons between groups where the variables were normally distributed, the Mann-Whitney U test was used for comparisons when the variables were nonnormal distribution, and Fisher’s exact test or Chi-squared test was used for comparisons when the variables were categorical.

The association between MAFLD and subclinical atherosclerosis was examined using multivariable logistic regression models in the cross-sectional study. The mixed-type random forest model was used to impute the missing data with the assessment of internally cross-validated error (29, 30). In model 1, the adjusted variables for comparison between MAFLD and non-MAFLD groups included age and sex. In model 2, we further adjusted leukocyte count, red blood cell count, platelet count, hemoglobin count, self-reported drinking, self-reported smoking, and comorbidities(coronary heart disease, CKD, stroke and cancer).

Cox proportional hazard regression models were used to evaluate the association between the presence of MAFLD and the development of SCA in the cohort. In model 1, the adjusted variables were age and sex. In model 2, we further adjusted leukocyte count, red blood cell count, platelet count, hemoglobin count, self-reported drinking, self-reported smoking, and CKD, *P* values < 0.05 were considered statistically significant.

P values for trend were calculated by treating HSI by tertile as ordinal in multivariable logistic regression models and Cox regression models. P values for the test of interaction for estimates from separate analyses were used to assess interaction by sex and age for each analysis (31). All reported P values are two sided and P <.05 was considered statistically significant.

### Sensitivity analyses

Three sensitivity analyses were conducted to confirm the reliability of the relation between baseline MAFLD and new-onset subclinical atherosclerosis in a longitudinal cohort. The sensitivity test I estimated the relation between MAFLD and new-onset SCA with follow-up over two years. The sensitivity test II adjusted FIB-4 to determine the relation between MAFLD and new-onset SCA. The sensitivity test III adjusted dyslipidemia to determine the relation between MAFLD and new-onset SCA.

## Result

### Baseline characteristics in the cross-sectional study

The baseline clinical and laboratory characteristics of the cross-sectional study are shown in **Table 1**. The mean (SD) age of study participants was 50.83 (10.68) in a total of 149,469 participants. Among them, 77,251 (51.68%) were diagnosed as MAFLD according to the definition. Compared with participants in the non-MAFLD group, participants in the MAFLD group were more likely to be males (71.02% vs. 45.58%), self-reported smokers (15.04% vs. 6.06%) and metabolically unhealthy, with higher BMI (26.95 vs. 23.30 kg/cm^2^), waist circumference (93.5 vs. 81.1 cm), FBG (6.04 vs. 5.36 mmol/L), SBP (130 vs. 122 mmHg), DBP (83 vs. 77 mmHg), LDL-c (3.09 vs. 2.89 mmol/L), TG (2.25 vs. 1.34 mmol/L) and lower level of HDL-c (1.16 vs. 1.40 mmol/L) (**Table 1**). Participants in the MAFLD group with higher HSI, were younger and metabolically unhealthy, with higher BMI, waist circumference, FBG, SBP, DBP, TC, TG, LDL-c and lower levels of HDL-c; and had more comorbidities, such as Type 2 diabetes, Hypertension, MetS, Dyslipidemia, Hyperuricaemia, CHD (**Table S1**).

**Table 1:** Baseline characteristics for the cross-sectional population.

	Total N=149,469	Non-MAFLD N=72,218	MAFLD N=77,251	*p value* ^a^
Age (year, mean (SD))	50.83 (10.68)	50.75 (11.77)	50.91 (9.56)	0.003
Gender, Female, n (%)	61691 (41.27)	39300 (54.42)	22391 (28.98)	<0.001
BMI (kg/m^2^, mean (SD))	25.19 (3.52)	23.30 (2.87)	26.95 (3.13)	<0.001
WC (cm, mean (SD))	88.6 (10.4)	81.1 (8.5)	93.5 (8.4)	<0.001
SBP (mmHg, mean (SD))	126 (19)	122 (19)	130 (18)	<0.001
DBP (mmHg, mean (SD))	80 (12)	77 (12)	83 (12)	<0.001
Self-reported smoking, n (%)	15996 (10.70)	4375 (6.06)	11621 (15.04)	<0.001
Self-reported drinking, n (%)	24747 (16.56)	7497 (10.38)	17250 (22.33)	<0.001
Laboratory Examination				
FBG (mmol/L, mean (SD))	5.71 (1.48)	5.36 (1.13)	6.04 (1.68)	<0.001
TC (mmol/L, mean (SD))	4.80 (0.95)	4.71 (0.91)	4.88 (0.97)	<0.001
TG (mmol/L, mean (SD))	1.81 (1.50)	1.34 (0.94)	2.25 (1.77)	<0.001
HDL-c (mmol/L, mean (SD))	1.28 (0.34)	1.40 (0.35)	1.16 (0.28)	<0.001
LDL-c (mmol/L, mean (SD))	3.00 (0.83)	2.89 (0.80)	3.09 (0.84)	<0.001
ALT (IU/L, mean (SD))	25.04 (21.40)	20.25 (20.88)	29.51 (20.90)	<0.001
AST (IU/L, mean (SD))	21.80 (12.30)	20.78 (12.85)	22.76 (11.68)	<0.001
BUN (mmol/L, mean (SD))	4.99 (1.34)	4.84 (1.40)	5.12 (1.28)	<0.001
Creatinine (μmol/L, mean (SD))	70.06 (19.26)	68.50 (21.91)	71.52 (16.28)	<0.001
Uric acid (μmol/L, mean (SD))	338.15 (89.59)	298.99 (78.90)	366.28 (86.19)	<0.001
LEU (×10^9^/L, mean (SD))	6.17 (1.65)	5.93 (1.62)	6.40 (1.64)	<0.001
RBC (×10^12^/L, mean (SD))	4.78 (0.47)	4.66 (0.47)	4.90 (0.44)	<0.001
HGB (g/L, mean (SD))	146.65 (15.61)	142.16 (16.07)	150.83 (13.93)	<0.001
PLT (×10^9^/L, mean (SD))	221.58 (54.61)	220.93 (55.67)	222.19 (53.59)	<0.001
Comorbidities				
Type 2 diabetes, n (%)	21490 (14.38)	5013 (6.94)	16477 (21.33)	<0.001
Hypertension, n (%)	52067 (34.83)	18456 (25.56)	33611 (43.51)	<0.001
MetS, n (%)	78895 (52.79)	21620 (29.94)	57275 (74.16)	<0.001
Dyslipidemia, n (%)	65723 (43.97)	21430 (29.67)	44293 (57.34)	<0.001
Hyperuricaemia, n (%)	18311 (12.30)	4149 (5.78)	14162 (18.40)	<0.001
CHD, n (%)	3114 (2.08)	1336 (1.85)	1778 (2.30)	<0.001
Cancer, n (%)	747 (0.50)	360 (0.50)	387 (0.50)	0.971
Stroke, n (%)	1337 (0.89)	535 (0.74)	802 (1.04)	<0.001
CKD, n (%)	1639 (1.11)	938 (1.31)	701 (0.91)	<0.001

MAFLD, metabolic dysfunction-associated fatty liver disease; SD, standard deviation; BMI, body mass index; WC, waist circumference; SBP, systolic blood pressure; DBP, diastolic blood pressure; FBG, fasting blood glucose; TC, total cholesterol; TG, triglycerides; LDL-C, low-density lipoprotein cholesterol; HDL-C, high-density lipoprotein cholesterol; ALT, alanine aminotransferase; AST, aspartate transaminase; BUN, blood urea nitrogen; LEU, leukocyte count; RBC, red blood cell; HGB, haemoglobin; PLT, platelet count; MetS, metabolic syndrome; CHD, coronary heart disease; CKD, chronic kidney disease.

a P values were calculated by student’s t-test for normally distributed variables and the Wilcoxon rank-sum test for non-normal distributed variables, as well as the chi-square test or Fisher’s exact test for categorical variables.

### Association of MAFLD and subclinical carotid atherosclerosis in the cross-sectional population

The proportions of SCA were higher in individuals in the MAFLD group compared to the non-MAFLD group (50.52% vs. 37.24%) (**Table 2**). The crude OR (95% CI) for SCA comparing by MAFLD was 1.72 (1.69-1.76; p < 0.001). After multivariable adjustment, SCA comparing individuals in the MAFLD group with those in the non-MAFLD group was 1.66 (95% CI 1.62-1.70; p<0.001). Furthermore, participants with higher levels of HSI had a greater risk of SCA after multivariable adjustments (**Table 2**). In the logistic regression analysis, compared with non-FLD, the ORs was 1.66 (95% CI 1.62-1.71, P < 0.001) for SCA with MAFLD,and 1.12 (95% CI, 1.02-1.24; P < 0.001) for SCA with FLD-nonMAFLD, respectively (**Table S2**). The participants with MAFLD had a 2.08-fold increased risk of increased CIMT (OR = 2.08; 95% CI = 2.02-2.14; p < 0.001), 1.53-fold increased risk of Carotid plaque (OR = 1.53; 95% CI = 1.49-1.57; p < 0.001) respectively after multivariable adjustments (**Table S3**).

**Table 2:** Association between MAFLD and subclinical carotid atherosclerosis in the cross-sectional analysis.

Groups	SCA events, n/N(%)	Odds ratio (95% confidence interval)								
		Crude	*p value* ^c^	*p for trend*	Model 1^a^	*p value* ^c^	*p for trend*	Model 2^b^	*p value* ^c^	*p for trend*
non-MAFLD Vs MAFLD										
non-MAFLD	26891/72218 (37.24)	Ref	–	–	Ref	–	–	Ref	–	–
MAFLD	39029/77251 (50.52)	1.72 (1.69,1.76)	<0.001		1.75 (1.70,1.79)	<0.001		1.66 (1.62,1.70)	<0.001	
non-MAFLD Vs MAFLD subgroups										
non-MAFLD	26891/72218 (37.24)	Ref	–	<0.001	Ref	–	<0.001	Ref	–	<0.001
MAFLD-lowHSI (HSI<35.63)	13278/25753 (51.56)	1.79 (1.74,1.85)	<0.001		1.49 (1.44,1.54)	<0.001		1.47 (1.42,1.52)	<0.001	
MAFLD-middleHSI (HSI:35.63-39.66)	13217/25749 (51.33)	1.78 (1.73,1.83)	<0.001		1.74 (1.69,1.80)	<0.001		1.66 (1.61,1.72)	<0.001	
MAFLD-highHSI (HSI>39.66)	12534/25749 (48.68)	1.60 (1.55,1.65)	<0.001		2.06 (2.00,2.13)	<0.001		1.88 (1.82,1.95)	<0.001	

MAFLD, metabolic dysfunction-associated fatty liver disease; SCA,Subclinical Carotid Atherosclerosis.

a Model 1 the adjustment factors included age and sex.

b Model 2 the adjustment factors included age, sex, self-reported smoking, self-reported drinking, red blood cell, leukocyte count, haemoglobin, platelet count, coronary heart disease, cancer, stroke and chronic kidney disease.

c P values were calculated based on Logistic regression.

### Baseline characteristics of the new-onset subclinical carotid atherosclerosis cohort

To explore the relationship between baseline MAFLD and new-onset SCA, we built a retrospective cohort that included participants who underwent multiple comprehensive health examinations at least one year apart. These individuals have performed abdominal and carotid ultrasound examinations at a Beijing health management center. The median follow-up period was 2.13 years (IQR: 1.58 - 3.02 years). The mean age was 46.61 years (SD, 7.07), and 32.17% of females at baseline. Compared with participants in the non-MAFLD group, participants in the MAFLD group had higher BMI, WC, BP, SCR, TG, TC, and UA, higher proportions with T2MD, hypertension, MetS, dyslipidemia, and hyperuricemia at baseline (all P < 0.01) (**Table 3**). Participants in the MAFLD group with higher HSI, were younger, metabolically unhealthy and more comorbidities (**Table S4**).

**Table 3:** Baseline characteristics for the longitudinal cohort.

	ALL N=2,179	Non-MAFLD N=849	MAFLD N=1,330	*p value* ^a^
Age (year, mean (SD))	46.61 (7.07)	45.69 (7.66)	47.21 (6.61)	<0.001
Gender, Female, n (%)	701 (32.17)	475 (55.95)	226 (16.99)	<0.001
BMI (kg/m^2^, mean (SD))	25.26 (3.38)	22.67 (2.44)	26.91 (2.82)	<0.001
WC (cm, mean (SD))	88.4 (10.6)	80.3 (8.7)	93.5 (8.3)	<0.001
SBP (mmHg, mean (SD))	116 (17)	109 (17)	121 (16)	<0.001
DBP (mmHg, mean (SD))	77 (11)	72 (10)	79 (11)	<0.001
Self-reported smoking, n (%)	41 (1.88)	7 (0.82)	34 (2.56)	0.003
Self-reported drinking, n (%)	89 (4.08)	20 (2.36)	69 (5.19)	0.001
Laboratory Examination				
FBG (mmol/L, mean (SD))	5.49 (1.17)	5.11 (0.72)	5.73 (1.33)	<0.001
TC (mmol/L, mean (SD))	4.72 (0.89)	4.60 (0.84)	4.79 (0.91)	<0.001
TG (mmol/L, mean (SD))	1.86 (1.74)	1.24 (0.93)	2.26 (2.01)	<0.001
HDL-c (mmol/L, mean (SD))	1.22 (0.34)	1.40 (0.35)	1.11 (0.28)	<0.001
LDL-c (mmol/L, mean (SD))	3.03 (0.77)	2.94 (0.75)	3.08 (0.78)	<0.001
ALT (IU/L, mean (SD))	25.40 (17.30)	18.10 (11.61)	30.06 (18.67)	<0.001
AST (IU/L, mean (SD))	20.59 (8.50)	18.26 (6.34)	22.07 (9.32)	<0.001
BUN (mmol/L, mean (SD))	4.95 (1.17)	4.74 (1.17)	5.08 (1.15)	<0.001
Creatinine (μmol/L, mean (SD))	67.94 (14.11)	64.00 (13.88)	70.46 (13.69)	<0.001
Uric acid (μmol/L, mean (SD))	346.11 (91.23)	298.25 (80.86)	376.71 (84.05)	<0.001
LEU (×10^9^/L, mean (SD))	5.84 (1.42)	5.55 (1.41)	6.03 (1.40)	<0.001
RBC (×10^12^/L, mean (SD))	4.77 (0.48)	4.57 (0.50)	4.89 (0.43)	<0.001
HGB (g/L, mean (SD))	145.45 (16.04)	138.21 (17.06)	150.06 (13.46)	<0.001
PLT (×10^9^/L, mean (SD))	222.33 (53.25)	226.62 (55.60)	219.61 (51.54)	0.003
Comorbidities				
Type 2 diabetes, n (%)	216 (9.91)	28 (3.30)	188 (14.14)	<0.001
Hypertension, n (%)	334 (15.33)	55 (6.48)	279 (20.98)	<0.001
MetS, n (%)	983 (45.15)	142 (16.75)	841 (63.28)	<0.001
Dyslipidemia, n (%)	931 (42.73)	186 (21.91)	745 (56.02)	<0.001
Hyperuricaemia, n (%)	320 (14.70)	43 (5.06)	277 (20.86)	<0.001
CHD, n (%)	0 (0)	0 (0)	0 (0)	–
Cancer, n (%)	0 (0)	0 (0)	0 (0)	–
Stroke, n (%)	0 (0)	0 (0)	0 (0)	–
CKD, n (%)	4 (0.18)	1 (0.12)	3 (0.23)	0.999

MAFLD, metabolic dysfunction-associated fatty liver disease; SD, standard deviation; BMI, body mass index; WC, waist circumference; SBP, systolic blood pressure; DBP, diastolic blood pressure; FBG, fasting blood glucose; TC, total cholesterol; TG, triglycerides; LDL-C, low-density lipoprotein cholesterol; HDL-C, high-density lipoprotein cholesterol; ALT, alanine aminotransferase; AST, aspartate transaminase; BUN, blood urea nitrogen; LEU, leukocyte count; RBC, red blood cell; HGB, haemoglobin; PLT, platelet count; MetS, metabolic syndrome; CHD, coronary heart disease; CKD, chronic kidney disease.

a P values were calculated by student’s t-test for normally distributed variables and the Wilcoxon rank-sum test for non-normal distributed variables, as well as the chi-square test or Fisher’s exact test for categorical variables.

### Association of the baseline MAFLD and the incidence subclinical carotid atherosclerosis in the longitudinal cohort

During the follow-up period, 849 participants developed SCA in 2,179 participants in the longitudinal cohort. In the multivariable-adjusted model, participants with baseline MAFLD had a higher risk of new-onset SCA, HR: 1.31 (95% CI 1.12-1.54, p=0.001). MAFLD groups had a greater risk of SCA after multivariable adjustments in the longitudinal cohort, HR 1.33 (95% CI 1.13,1.57). However, compared with non-FLD, FLD-nonMAFLD group is not associated with a high risk of SCA occurring, HR 1.35 (95% CI 0.75-2.42; p = 0.312) (**Table S5**). After multivariable-adjusted, increased CIMT, Carotid plaque comparing individuals in the MAFLD group with those in the non-MAFLD group were 1.38 (95% CI 1.15-1.66; p<0.001) and 1.31 (95% CI 1.04-1.65; p=0.024) (**Table S6**). Consistent with cross-sectional studies, the correlations between baseline MAFLD hepatic steatosis level and the progression of SCA were described in **Table 4**. Compared with the Non-MAFLD group, participants in the MAFLD group with higher hepatic steatosis levels at baseline had a higher risk of developing SCA after adjustment for confounders (**Table 4**).

**Table 4:** Association between baseline MAFLD and the incidence of subclinical carotid atherosclerosis in the longitudinal cohort.

Groups	SCA events, n/N(%)	Hazards ratio (95% confidence interval)								
		Crude	*p value* ^c^	*p for trend*	Model 1^a^	*p value* ^c^	*p for trend*	Model 2^b^	*p value* ^c^	*p for trend*
non-MAFLD Vs MAFLD										
non-MAFLD	243/849 (28.62)	Ref	–	–	Ref	–	–	Ref	–	–
MAFLD	642/1330 (48.27)	1.51 (1.30,1.75)	<0.001		1.30 (1.11,1.53)	0.001		1.31 (1.12,1.54)	0.001	
**non-MAFLD Vs** MAFLD **subgroups**										
non-MAFLD	243/849 (28.62)	Ref	–	<0.001	Ref	–	0.001	Ref	–	<0.001
MAFLD-lowHSI (HSI<35.63)	208/464 (44.83)	1.49 (1.24,1.80)	<0.001		1.22 (1.00,1.48)	0.044		1.23 (1.02,1.50)	0.035	
MAFLD-middleHSI (HSI:35.63-39.66)	211/438 (48.17)	1.56 (1.30,1.88)	<0.001		1.33 (1.10,1.62)	0.004		1.34 (1.10,1.63)	0.003	
MAFLD-highHSI (HSI>39.66)	223/428 (52.10)	1.48 (1.23,1.77)	<0.001		1.36 (1.12,1.65)	0.002		1.38 (1.14,1.68)	0.001	

MAFLD, metabolic dysfunction-associated fatty liver disease; SCA,Subclinical Carotid Atherosclerosis.

a Model 1 the adjustment factors included age and sex.

b Model 2 the adjustment factors included age, sex, self-reported smoking, self-reported drinking, red blood cell, leukocyte count, haemoglobin, platelet count and chronic kidney disease.

c P values were calculated based on Cox regression.

Subgroup analysis according to sex and age for baseline MAFLD and new-onset SCA were shown in **Table 5**. After multivariable-adjusted, the HR (95% CI) of new-onset SCA comparing non-MAFLD group to MAFLD group for female and male was 1.46 (1.09-1.95; p = 0.011) and 1.24 (1.02-1.50; p = 0.029) respectively. At follow-up visit, participants with MAFLD younger than 45 years old had a 1.67-fold increased risk of new-onset SCA (HR = 1.67; 95% CI = 1.17-2.40; p = 0.005), which were higher than a 1.21-fold risk of incident SCA in MAFLD individuals over 45 years of age (HR = 1.21; 95% CI = 1.01–1.45; p = 0.041). Interestingly, we found a significant interaction between MAFLD and age (p for interaction = 0.034).

**Table 5:** Association between baseline MAFLD and the subclinical carotid atherosclerosis in the cohort subgroups.

Subgroups	Groups	SCA events, n/N (%)	Hazards ratio (95% confidence interval)						
			Crude	*p value^c^*	Model 1^a^	*p value^c^*	Model 2^b^	*p value^c^*	*p for interaction^d^*
Sex subgroups									
**Female**	non-MAFLD	112/475 (23.58)	Ref	–	Ref	–	Ref	–	0.304
	MAFLD	92/226 (40.71)	1.72 (1.31,2.27)	<0.001	1.41 (1.06,1.88)	0.018	1.46 (1.09,1.95)	0.011	
**Male**	non-MAFLD	131/374 (35.03)	Ref	–	Ref	–	Ref	–	
	MAFLD	550/1104 (49.82)	1.24 (1.02,1.50)	0.029	1.24 (1.03,1.50)	0.026	1.24 (1.02,1.50)	0.029	
Age subgroups									
**Age<45**	non-MAFLD	48/351 (13.68)	Ref	–	Ref	–	Ref	–	0.034
	MAFLD	170/427 (39.81)	2.20 (1.59,3.04)	<0.001	1.72 (1.20,2.46)	0.003	1.67 (1.17,2.40)	0.005	
**Age≥45**	non-MAFLD	195/498 (39.16)	Ref	–	Ref	–	Ref	–	
	MAFLD	472/903 (52.27)	1.31 (1.11,1.55)	0.001	1.18 (0.99,1.41)	0.072	1.21 (1.01,1.45)	0.041	

MAFLD, metabolic dysfunction-associated fatty liver disease.

a Model 1 the adjustment factors included age and sex.

b Model 2 the adjustment factors included age, sex, self-reported smoking, self-reported drinking, red blood cell, leukocyte count, haemoglobin, platelet count and chronic kidney disease.

c P values were calculated based on Cox regression.

d P for interaction were calculated based on Cox regression and the adjustment factors included age, sex, self-reported smoking, self-reported drinking, red blood cell, leukocyte count, haemoglobin, platelet count and chronic kidney disease.

### Sensitivity analysis

Our findings in longitudinal studies were robust to the following sensitivity analyses. First, MAFLD was associated with the development of SCA in the cohort with a follow-up time of > 2 years (**Table S7**). Second, the association between baseline MAFLD and new-onset SCA persisted after further adjusted FIB-4 in Cox proportional hazard regression model (**Table S8**). Last, considering the influence of lipid abnormalities on SCA, we further adjusted dyslipidemia in the Cox proportional hazard regression model (**Table S9**).

## Discussion

In this study of 153,482 participants from health examination centers, we found that MAFLD was significantly associated with SCA after adjusting for potential confounders. And the association was progressive increased with the categories of hepatic steatosis index. In a Beijing longitudinal population, we found that participants with MAFLD had a high risk of SCA developing compared to those without MAFLD. The risk of developing SCA was also higher in participants with severe hepatic steatosis in the longitudinal study. Interaction with age should be mentioned. Furthermore, this association was present across genders and ages and was more pronounced in males and young adults (aged < 45 years old). Thus, Our findings suggested that MAFLD may be a risk factor for carotid atherosclerosis development and the impacts from MAFLD are more pronounced in younger populations or those with severe hepatic steatosis.

NAFLD is considered a demonstration of multisystem metabolic dysfunction, with insulin resistance as a common pathophysiological mechanism (32, 33). NAFLD has been underestimated as an independent risk factor for ASCVD after adjustment for conventional ASCVD risk factors in a large number of studies (34, 35). The correlation between NAFLD and the presence and occurrence of SCA has been established by several previous studies and meta-analyses (11, 28, 36–38). As a new definition proposed by an international panel of experts, MAFLD has been demonstrated to have superior utility in clinical practice by multiple studies from the United States and Japan, to identify high-risk groups for early intervention for diseases such as fibrosis and metabolic disorders and chronic kidney disease (39, 40). Results from a community-based cohort study from Sri Lanka showed that subjects excluded by the NAFLD definition but captured by the MAFLD definition were significantly more likely to be excluded by MAFLD definition but captured by the NAFLD definition than subjects excluded by MAFLD definition but captured by the NAFLD definition, new-onset metabolic characteristics and cardiovascular events were higher (41). Two cohort studies from the UK and Korea confirmed that this MAFLD was independently associated with an increased risk of intrahepatic and extrahepatic events. In the non-Chinese population, participants with MAFLD were reported to be at higher risk of CVD than participants with NAFLD (42, 43). A cross-sectional study based on participants enrolled in East China demonstrated that individuals with MAFLD have a similar or higher risk of future CVD than those with NAFLD (44). A recent cross-sectional study and a community-based cohort study also reported positive correlations between MAFLD and subclinical atherosclerosis measured by CIMT, brachial-ankle pulse wave velocity, microalbuminuria and coronary artery calcification score (12, 45). A matched case-control study that included 3306 patients from China showed that MAFLD was significantly associated with the risk of MACEs in patients with chronic coronary syndrome (46). However, NAFLD/MAFLD still haven’t received sufficient attention from the Cardiology community (47). The association between MAFLD and CVD/SCA is mainly attributed to their common risk factors, such as obesity and lipid abnormalities (48). MAFLD highlights the systemic metabolic disorders that accompany fatty liver disease. Therefore, it is necessary to further explore the relationship between MAFLD and SCA development in a large Chinese population. In this study, the correlation between MAFLD and SCA detected using ultrasound was observed in a nationwide health examination population in China. Furthermore, among a retrospective cohort population who underwent at least two comprehensive health check-up examinations in Beijing, the risk of the development of carotid atherosclerosis in participants with MAFLD was significantly higher than in participants without MAFLD.

In recent years, numerous population-based studies have also shown that the severity of liver fibrosis in NAFLD patients is closely associated with increased CIMT (2, 49, 50). However, the impact of steatosis is less clear and controversial. Liver fibrosis is the manifestation of advanced NAFLD after a long-term metabolic disorder. It might not be conducive to the early intervention of the disease that correlates with subclinical atherosclerosis and liver fibrosis. If the correlation between early lipogenesis and atherosclerosis is established, early intervention of lipogenesis may be beneficial for early intervention of the disease. Therefore, fibrosis is considered the most important factor for identifying risk stratification in NAFLD (51). However, this may mask the risk of quality of life and early subclinical outcomes in patients with steatosis. In this study, we observed the association of MAFLD and the presence and occurrence of SCA were progressive across categories of hepatic steatosis index, which showed a significant correlation with fatty liver grade (21).

Furthermore, in the sensitivity analysis of this study, we further adjusted FIB-4 and found that the relationship between MAFLD and SCA still existed. Interestingly, we first observed a higher risk of developing SCA in the younger MAFLD population. However, well-designed prospective studies are warranted to elucidate this relationship between carotid atherosclerosis and MAFLD in this patient subgroup.

There are pathological mechanisms linking MAFLD and atherosclerosis. Insulin resistance and systematical inflammation were considered key mediators in this association (52–54). In the patient with insulin resistance, the activity of various lipases is disturbed, leading to an excessive influx of free fatty acids and the intestinal production of chylomicrons (CMs) and VLDL to the liver. Hyperinsulinemia also leads to increased esterification of fatty acids rather than β-oxidation, resulting in the formation of TGs in hepatocytes. Patients with metabolic disorders have increased systematic oxidative stress and glucose levels, and excessive circulating LDL levels can be further modified to generate large amounts of ox-LDL and glycated-LDL, which lead to foam cell formation and atherosclerotic disease (4). Recently, a study revealed that nicotine accumulates in the gut during smoking and promotes NASH progression through the gut-liver dialogue (55). In fact, as the most common unhealthy behaviours among humans, Smoking may be a risk factor shared by CVD and MAFLD, dur to studies have shown that smokers have a higher risk of insulin resistance and hyperinsulinemia compared with non-smokers. And smoking could altered lipid metabolism by increasing lipolysis, insulin resistance, and tissue lipotoxicity (56). The fatty hepatocyte in NAFLD is inevitably accompanied by mitochondrial dysfunction and the occurrence of hepatocyte degeneration and necrosis, inducing the production of inflammatory chemicals such as c-reactive protein, tumor necrosis factors α, interleukin-6, interleukin-12 and monocyte chemoattractant protein-1 in hepatocytes, leading to an inflammatory response in the liver, and systemic low-grade inflammatory response. These inflammatory mediators play synergistic and antagonistic roles in the initiation and progression of arteriosclerosis diseases (57, 58). In addition, adiponectin concentration, oxidative stress, and endothelial dysfunction in NAFLD patients also contribute to the development of atherosclerosis (2, 59–61). Currently, there is no consensus on how NAFLD steatosis degree affects atherosclerosis. The more severe lipotoxicity, inflammation, and marked hepatic insulin resistance in the progression of hepatic steatosis may partially explain the relationship between the severity of hepatic steatosis and atherosclerosis (62). A population study which provided ideas for early warning of SCA risk in MAFLD patients found that cystine, sphingomyelin (16:1/18:1), *de novo* lipogenesis (16:0/18:2n-6) were significant associated with SCA in obese MAFLD patients and phosphatidylethanolamine (PE 20:2/16:0), phosphatidylglycerol (PG 18:0/20:4), *de novo* lipogenesis (16:0/18:2n-6) were significant associated with SCA were significant associated with SCA in nonobese MAFLD patients (63).

There are several limitations to consider when interpreting our findings. First, our study is based on the national health screening population, which is not a random sample and may not represent the Chinese rural population. Second, retrospective study designs can lead to unavoidable biases, including selection or misclassification bias. Third, due to the long duration of the study, different ultrasound scanners and radiological technologists were involved in FLD measurements over time. However, the researchers who collected the data did not know the purpose of the study, and equipment changes were not related to participant characteristics. Finally, our study was conducted in a population with regular health checks, and these findings may not generalize to other populations, especially different age or racial/ethnic groups.

## Conclusion

In conclusion, our study showed that MAFLD had an increased risk of the presence and development of SCA in cross-sectional and longitudinal populations. The risk of SCA was higher among individuals with more advanced MAFLD steatosis and the younger population. This study provides critical evidence for the risks of existing and emerging SCA in MAFLD. It highlights the importance of early interventions in MAFLD in young adults and patients with severe steatosis to prevent clinical atherosclerosis. Prospective studies and clinical trials focusing on whether the management of MAFLD can result in a reduced risk of cardiovascular disease are warranted.

## Data availability statement

The original contributions presented in the study are included in the article/**Supplementary Material**. Further inquiries can be directed to the corresponding authors.

## Ethics statement

The study was in accordance with the principles of the Declaration of Helsinki and was approved by the central ethics board of Renmin Hospital of Wuhan University, followed by the acceptance with the ethics center in each collaborating hospital. Individual identification data was removed, and only anonymous information was kept during the study process. The ethics committees from each hospital waived patient informed consent.

## Author contributions

FL, X-MW and CW designed the study, collected and analyzed data and drafted the manuscript. XH, Y-ML and J-JQ collected and interpreted data and contributed to data analysis. PZ, Y-XJ, Z-GS and JC performed critical revision of the manuscript for important intellectual content. H-PL, X-JZ and HL conceived and supervised the study, critical revision of the manuscript for important intellectual content, and supervised the study. All authors approved the paper for submission.

## References

[1] Cai J Xu M Zhang X Li H. Innate immune signaling in nonalcoholic fatty liver disease and cardiovascular diseases. Annu Rev Pathol (2019) 14:153–84. doi: 10.1146/annurev-pathmechdis-012418-013003 30230967

[2] Li W Liu J Cai J Zhang XJ Zhang P She ZG. NAFLD as a continuous driver in the whole spectrum of vascular disease. J Mol Cell Cardiol (2022) 163:118–32. doi: 10.1016/j.yjmcc.2021.10.007 34737121

[3] Zhou J Bai L Zhang XJ Li H Cai J. Nonalcoholic fatty liver disease and cardiac remodeling risk: Pathophysiological mechanisms and clinical implications. Hepatology (2021) 74(5):2839–47. doi: 10.1002/hep.32072 34309877

[4] Cai J Zhang XJ Ji YX Zhang P She ZG Li H. Nonalcoholic fatty liver disease pandemic fuels the upsurge in cardiovascular diseases. Circ Res (2020) 126(5):679–704. doi: 10.1161/CIRCRESAHA.119.316337 32105577

[5] Zhou J Zhou F Wang W Zhang XJ Ji YX Zhang P. Epidemiological features of NAFLD from 1999 to 2018 in China. Hepatology (2020) 71(5):1851–64. doi: 10.1002/hep.31150 32012320

[6] Zhou F Zhou J Wang W Zhang XJ Ji YX Zhang P. Unexpected rapid increase in the burden of NAFLD in China from 2008 to 2018: A systematic review and meta-analysis. Hepatology (2019) 70(4):1119–33. doi: 10.1002/hep.30702 31070259

[7] Lei F Qin J Song X Liu Y Chen M Sun T. The prevalence of MAFLD and its association with atrial fibrillation in a nationwide health check-up population in China. Front Endocrinol (2022) 13:1007171. doi: 10.3389/fendo.2022.1007171 PMC9551383 36237179

[8] Lester SJ Eleid MF Khandheria BK Hurst RT. Carotid intima-media thickness and coronary artery calcium score as indications of subclinical atherosclerosis. Mayo Clin Proc (2009) 84(3):229–33. doi: 10.1016/S0025-6196(11)61139-7 PMC2664607 19252109

[9] Lorenz MW Markus HS Bots ML Rosvall M Sitzer M. Prediction of clinical cardiovascular events with carotid intima-media thickness: A systematic review and meta-analysis. Circulation (2007) 115(4):459–67. doi: 10.1161/CIRCULATIONAHA.106.628875 17242284

[10] Targher G Bertolini L Padovani R Zenari L Zoppini G Falezza G. Relation of nonalcoholic hepatic steatosis to early carotid atherosclerosis in healthy men: Role of visceral fat accumulation. Diabetes Care (2004) 27(10):2498–500. doi: 10.2337/diacare.27.10.2498 15451925

[11] Oni ET Agatston AS Blaha MJ Fialkow J Cury R Sposito A. A systematic review: Burden and severity of subclinical cardiovascular disease among those with nonalcoholic fatty liver; should we care? Atherosclerosis (2013) 230(2):258–67. doi: 10.1016/j.atherosclerosis.2013.07.052 24075754

[12] Liu S Wang J Wu S Niu J Zheng R Bie L. The progression and regression of metabolic dysfunction-associated fatty liver disease are associated with the development of subclinical atherosclerosis: A prospective analysis. Metabolism (2021) 120:154779. doi: 10.1016/j.metabol.2021.154779 33895182

[13] Lu Y Pechlaner R Cai J Yuan H Huang Z Yang G. Trajectories of age-related arterial stiffness in chinese men and women. J Am Coll Cardiol (2020) 75(8):870–80. doi: 10.1016/j.jacc.2019.12.039 32130922

[14] Cao X Chen Z Wu L Zhou J. Co-Occurrence of chronic pain, depressive symptoms, and poor sleep quality in a health check-up population in china:a multicenter survey. J Affect Disord (2021) 281:792–8. doi: 10.1016/j.jad.2020.11.060 33229026

[15] China Medical and health culture association-medical and health credit branch. White paper on trend insights of china’s comprehensive health management service industry. (2021).

[16] Chinese Elderly Type 2 Diabetes Prevention and Treatment of Clinical Guidelines Writing Group Geriatric Endocrinology and Metabolism Branch of Chinese Geriatric Society Geriatric Endocrinology and Metabolism Branch of Chinese Geriatric Health Care Society Geriatric Professional Committee of Beijing Medical Award Foundation National Clinical Medical Research Center for Geriatric Diseases (PLA General Hospital). Clinical guidelines for prevention and treatment of type 2 diabetes mellitus in the elderly in China (2022 edition). Zhonghua Nei Ke Za Zhi (2022) 61(1):12–50. doi: 10.3760/cma.j.cn112138-20211027-00751 34979769

[17] Joint Committee for Guideline Revision 2018 chinese guidelines for prevention and treatment of hypertension-a report of the revision committee of chinese guidelines for prevention and treatment of hypertension. J Geriatr. Cardiol (2019) 16(3):182–241. doi: 10.11909/j.issn.1671-5411.2019.03.014 31080465 PMC6500570

[18] Joint Committee for Developing Chinese guidelines on Prevention and Treatment of Dyslipidemia in Adults Chinese Guidelines on prevention and treatment of dyslipidemia in adults. Zhonghua Xin Xue Guan Bing Za Zhi (2007) 35(5):390–419. 17711682

[19] Borghi C Domienik-Karlowicz J Tykarski A Widecka K Filipiak KJ Jaguszewski MJ. Expert consensus for the diagnosis and treatment of patient with hyperuricemia and high cardiovascular risk: 2021 update. Cardiol J (2021) 28(1):1–14. doi: 10.5603/CJ.a2021.0001 33438180 PMC8105060

[20] Alberti KG Eckel RH Grundy SM Zimmet PZ Cleeman JI Donato KA. Harmonizing the metabolic syndrome: A joint interim statement of the international diabetes federation task force on epidemiology and prevention; national heart, lung, and blood institute; American heart association; world heart federation; international atherosclerosis society; and international association for the study of obesity. Circulation (2009) 120(16):1640–5. doi: 10.1161/CIRCULATIONAHA.109.192644 19805654

[21] Lee JH Kim D Kim HJ Lee CH Yang JI Kim W. Hepatic steatosis index: A simple screening tool reflecting nonalcoholic fatty liver disease. Dig Liver Dis (2010) 42(7):503–8. doi: 10.1016/j.dld.2009.08.002 19766548

[22] European Association for the Study of the Liver (EASL) European Association for the Study of Diabetes (EASD) European Association for the Study of Obesity (EASO) EASL-EASD-EASO clinical practice guidelines for the management of non-alcoholic fatty liver disease. J Hepatol (2016) 64(6):1388–402. doi: 10.1016/j.jhep.2015.11.004 27062661

[23] Ma YC Zuo L Chen JH Luo Q Yu XQ Li Y. Modified glomerular filtration rate estimating equation for Chinese patients with chronic kidney disease. J Am Soc Nephrol. (2006) 17(10):2937–44. doi: 10.1681/ASN.2006040368 16988059

[24] National Kidney Foundation. K/DOQI clinical practice guidelines for chronic kidney disease: Evaluation, classification, and stratification. Am J Kidney Dis (2002) 39(2 Suppl 1):S1–266. 11904577

[25] Guo B Guo Y Nima Q Feng Y Wang Z Lu R. Exposure to air pollution is associated with an increased risk of metabolic dysfunction-associated fatty liver disease. J Hepatol (2022) 76(3):518–25. doi: 10.1016/j.jhep.2021.10.016 34883157

[26] Dasarathy S Dasarathy J Khiyami A Joseph R Lopez R McCullough AJ. Validity of real time ultrasound in the diagnosis of hepatic steatosis: A prospective study. J Hepatol (2009) 51(6):1061–7. doi: 10.1016/j.jhep.2009.09.001 PMC6136148 19846234

[27] National Workshop on Fatty Liver and Alcoholic Liver Disease Chinese Society of Hepatology Chinese Medical Association Fatty Liver Expert Committee Chinese Medical Doctor Association. Guidelines of prevention and treatment for nonalcoholic fatty liver disease: A 2018 update. Zhonghua Gan Zang Bing Za Zhi (2018) 26(3):195–203. doi: 10.3760/cma.j.issn.1007-3418.2018.03.008 29804393 PMC12769340

[28] Sinn DH Cho SJ Gu S Seong D Kang D Kim H. Persistent nonalcoholic fatty liver disease increases risk for carotid atherosclerosis. Gastroenterology (2016) 151(3):481–488.e1. doi: 10.1053/j.gastro.2016.06.001 27283259

[29] Zhu L She ZG Cheng X Qin JJ Zhang XJ Cai J. Association of blood glucose control and outcomes in patients with COVID-19 and pre-existing type 2 diabetes. Cell Metab (2020) 31(6):1068–1077.e3. doi: 10.1016/j.cmet.2020.04.021 32369736 PMC7252168

[30] Waljee AK Mukherjee A Singal AG Zhang Y Warren J Balis U. Comparison of imputation methods for missing laboratory data in medicine. BMJ Open (2013) 3(8):e002847. doi: 10.1136/bmjopen-2013-002847 PMC3733317 23906948

[31] Altman DG Bland JM. Interaction revisited: The difference between two estimates. BMJ (2003) 326(7382):219. doi: 10.1136/bmj.326.7382.219 12543843 PMC1125071

[32] Cohen JC Horton JD Hobbs HH. Human fatty liver disease: Old questions and new insights. Science (2011) 332(6037):1519–23. doi: 10.1126/science.1204265 PMC3229276 21700865

[33] Than NN Newsome PN. A concise review of non-alcoholic fatty liver disease. Atherosclerosis (2015) 239(1):192–202. doi: 10.1016/j.atherosclerosis.2015.01.001 25617860

[34] Sao R Aronow WS. Association of non-alcoholic fatty liver disease with cardiovascular disease and subclinical atherosclerosis. Arch Med Sci (2018) 14(6):1233–44. doi: 10.5114/aoms.2017.68821 PMC6209727 30393477

[35] Stefan N Haring HU Cusi K. Non-alcoholic fatty liver disease: Causes, diagnosis, cardiometabolic consequences, and treatment strategies. Lancet Diabetes Endocrinol (2019) 7(4):313–24. doi: 10.1016/S2213-8587(18)30154-2 30174213

[36] Mellinger JL Pencina KM Massaro JM Hoffmann U Seshadri S Fox CS. Hepatic steatosis and cardiovascular disease outcomes: An analysis of the framingham heart study. J Hepatol (2015) 63(2):470–6. doi: 10.1016/j.jhep.2015.02.045 PMC5282653 25776891

[37] Ozturk K Uygun A Guler AK Demirci H Ozdemir C Cakir M. Nonalcoholic fatty liver disease is an independent risk factor for atherosclerosis in young adult men. Atherosclerosis (2015) 240(2):380–6. doi: 10.1016/j.atherosclerosis.2015.04.009 25875390

[38] Buckley AJ Thomas EL Lessan N Trovato FM Trovato GM Taylor-Robinson SD. Non-alcoholic fatty liver disease: Relationship with cardiovascular risk markers and clinical endpoints. Diabetes Res Clin Pract (2018) 144:144–52. doi: 10.1016/j.diabres.2018.08.011 30170074

[39] Lin S Huang J Wang M Kumar R Liu Y Liu S. Comparison of MAFLD and NAFLD diagnostic criteria in real world. Liver Int (2020) 40(9):2082–9. doi: 10.1111/liv.14548 32478487

[40] Yamamura S Eslam M Kawaguchi T Tsutsumi T Nakano D Yoshinaga S. MAFLD identifies patients with significant hepatic fibrosis better than NAFLD. Liver Int (2020) 40(12):3018–30. doi: 10.1111/liv.14675 32997882

[41] Niriella MA Ediriweera DS Kasturiratne A De Silva ST Dassanayaka AS De Silva AP. Outcomes of NAFLD and MAFLD: Results from a community-based, prospective cohort study. PloS One (2021) 16(2):e0245762. doi: 10.1371/journal.pone.0245762 33534815 PMC7857550

[42] Lee H Lee YH Kim SU Kim HC. Metabolic dysfunction-associated fatty liver disease and incident cardiovascular disease risk: A nationwide cohort study. Clin Gastroenterol Hepatol (2021) 19(10):2138–2147.e10. doi: 10.1016/j.cgh.2020.12.022 33348045

[43] Yoneda M Yamamoto T Honda Y Imajo K Ogawa Y Kessoku T. Risk of cardiovascular disease in patients with fatty liver disease as defined from the metabolic dysfunction associated fatty liver disease or nonalcoholic fatty liver disease point of view: A retrospective nationwide claims database study in Japan. J Gastroenterol (2021) 56(11):1022–32. doi: 10.1007/s00535-021-01828-6 PMC8531127 34601620

[44] Wang Y Yu Y Zhang H Chen C Wan H Chen Y. Cardiovascular and renal burdens among patients with MAFLD and NAFLD in China. Front Endocrinol (Lausanne) (2022) 13:968766. doi: 10.3389/fendo.2022.968766 36120461 PMC9480613

[45] Bessho R Kashiwagi K Ikura A Yamataka K Inaishi J Takaishi H. A significant risk of metabolic dysfunction-associated fatty liver disease plus diabetes on subclinical atherosclerosis. PloS One (2022) 17(5):e0269265. doi: 10.1371/journal.pone.0269265 35639744 PMC9154100

[46] Liu HH Cao YX Jin JL Guo YL Zhu CG Wu NQ. Metabolic-associated fatty liver disease and major adverse cardiac events in patients with chronic coronary syndrome: A matched case-control study. Hepatol Int (2021) 15(6):1337–46. doi: 10.1007/s12072-021-10252-0 34626331

[47] Zhou XD Cai J Targher G Byrne CD Shapiro MD Sung KC. Metabolic dysfunction-associated fatty liver disease and implications for cardiovascular risk and disease prevention. Cardiovasc Diabetol (2022) 21(1):270. doi: 10.1186/s12933-022-01697-0 36463192 PMC9719631

[48] Miptah HN Ramli AS Mohamad M Hashim H Tharek Z. Non-alcoholic fatty liver disease (NAFLD) and the cardiovascular disease (CVD) risk categories in primary care: Is there an association? BMC Fam. Pract (2020) 21(1):238. doi: 10.1186/s12875-020-01306-7 33218301 PMC7679975

[49] Chen Y Xu M Wang T Sun J Sun W Xu B. Advanced fibrosis associates with atherosclerosis in subjects with nonalcoholic fatty liver disease. Atherosclerosis (2015) 241(1):145–50. doi: 10.1016/j.atherosclerosis.2015.05.002 25988358

[50] Xin Z Zhu Y Wang S Liu S Xu M Wang T. Associations of subclinical atherosclerosis with nonalcoholic fatty liver disease and fibrosis assessed by non-invasive score. Liver Int (2020) 40(4):806–14. doi: 10.1111/liv.14322 31820847

[51] Ekstedt M Hagstrom H Nasr P Fredrikson M Stal P Kechagias S. Fibrosis stage is the strongest predictor for disease-specific mortality in NAFLD after up to 33 years of follow-up. Hepatology (2015) 61(5):1547–54. doi: 10.1002/hep.27368 25125077

[52] Tilg H Effenberger M. From NAFLD to MAFLD: When pathophysiology succeeds. Nat Rev Gastroenterol Hepatol (2020) 17(7):387–8. doi: 10.1038/s41575-020-0316-6 32461575

[53] Villela-Nogueira CA Leite NC Cardoso CR Salles GF. NAFLD and increased aortic stiffness: Parallel or common physiopathological mechanisms? int. J Mol Sci (2016) 17(4):460. doi: 10.3390/ijms17040460 PMC4848916 27104526

[54] Gaggini M Morelli M Buzzigoli E DeFronzo RA Bugianesi E Gastaldelli A. Non-alcoholic fatty liver disease (NAFLD) and its connection with insulin resistance, dyslipidemia, atherosclerosis and coronary heart disease. Nutrients (2013) 5(5):1544–60. doi: 10.3390/nu5051544 PMC3708335 23666091

[55] Chen B Sun L Zeng G Shen Z Wang K Yin L. Gut bacteria alleviate smoking-related NASH by degrading gut nicotine. Nature (2022) 610(7932):562–8. doi: 10.1038/s41586-022-05299-4 PMC9589931 36261549

[56] Gastaldelli A Folli F Maffei S. Impact of tobacco smoking on lipid metabolism, body weight and cardiometabolic risk. Curr Pharm Des (2010) 16(23):2526–30. doi: 10.2174/138161210792062858 20550509

[57] Bieghs V Rensen PC Hofker MH Shiri-Sverdlov R. NASH and atherosclerosis are two aspects of a shared disease: Central role for macrophages. Atherosclerosis (2012) 220(2):287–93. doi: 10.1016/j.atherosclerosis.2011.08.041 21930273

[58] Lee YJ Shim JY Moon BS Shin YH Jung DH Lee JH. The relationship between arterial stiffness and nonalcoholic fatty liver disease. Dig Dis Sci (2012) 57(1):196–203. doi: 10.1007/s10620-011-1819-3 21750929

[59] Villanova N Moscatiello S Ramilli S Bugianesi E Magalotti D Vanni E. Endothelial dysfunction and cardiovascular risk profile in nonalcoholic fatty liver disease. Hepatology (2005) 42(2):473–80. doi: 10.1002/hep.20781 15981216

[60] Alharthi J Latchoumanin O George J Eslam M. Macrophages in metabolic associated fatty liver disease. World J Gastroenterol (2020) 26(16):1861–78. doi: 10.3748/wjg.v26.i16.1861 PMC7201150 32390698

[61] Polimeni L Del BM Baratta F Perri L Albanese F Pastori D. Oxidative stress: New insights on the association of non-alcoholic fatty liver disease and atherosclerosis. World J Hepatol (2015) 7(10):1325–36. doi: 10.4254/wjh.v7.i10.1325 PMC4450196 26052378

[62] Lyu K Zhang Y Zhang D Kahn M Ter Horst KW Rodrigues M. A membrane-bound diacylglycerol species induces PKC-mediated hepatic insulin resistance. Cell Metab (2020) 32(4):654–664.e5. doi: 10.1016/j.cmet.2020.08.001 32882164 PMC7544641

[63] Shao C Xu L Lei P Wang W Feng S Ye J. Metabolomics to identify fingerprints of carotid atherosclerosis in nonobese metabolic dysfunction-associated fatty liver disease. J Transl Med (2023) 21(1):12. doi: 10.1186/s12967-022-03760-6 36624524 PMC9830861

