# Supplementary material for: Metabolic dysfunction-associated fatty liver disease increased the risk of subclinical carotid atherosclerosis in China
Source: Front Endocrinol (Lausanne). 2023 Apr 4;14:1109673. doi: 10.3389/fendo.2023.1109673 (PMC10110917; doi:10.3389/fendo.2023.1109673)
Supplement: Supplementary file 1 [file Table_1.docx]

**Supplemental information: Metabolic Dysfunction-Associated Fatty Liver Disease increased the risk of subclinical carotid atherosclerosis in China**

**Supplemental Table list:**

**Table S1 Baseline Characteristics for the MAFLD HSI Subgroups in the Longitudinal Cohort**

**Table S2 Association between nonFLD/FLD-nonMAFLD/MAFLD and Subclinical Carotid Atherosclerosis in the Cross-sectional Analysis**

**Table S3 Association between MAFLD and elevated Carotid Intima-media Thickness/Carotid Plaque in the Cross-sectional Analysis**

**Table S4 Baseline Characteristics for the MAFLD HSI Subgroups in the Longitudinal Cohort**

**Table S5 Association between nonFLD/FLD-nonMAFLD/MAFLD and Subclinical Carotid Atherosclerosis in the Longitudinal Cohort**

**Table S6 Association between MAFLD and elevated Carotid Intima-media Thickness/Carotid Plaque in the Longitudinal Cohort**

**Table S7 Sensitivity Test I for the Incidence of Subclinical Carotid Atherosclerosis in MAFLD group versus non-MAFLD group during Follow-up Time above 2 Years**

**Table S8 Sensitivity Test II for the Incidence of Subclinical Carotid Atherosclerosis in MAFLD Group versus non-MAFLD Group after further adjusting FIB-4**

**Table S9 Sensitivity Test III for the incidence of Subclinical Carotid Atherosclerosis in MAFLD Group versus Non-MAFLD Group after further adjusting Dyslipidemia**

**Table S1 Baseline Characteristics for the MAFLD HSI Subgroups in the Longitudinal Cohort**

|  | **MAFLD-lowHSI (HSI<35.63)** | **MAFLD-middleHSI (HSI:35.63-39.66)** | **MAFLD-highHSI (HSI>39.66)** |
| --- | --- | --- | --- |
|  | **N=25,753** | **N=25,749** | **N=25,749** |
| Age (year, mean (SD)) | 52.82(9.60) | 51.27(9.19) | 48.64(9.40) |
| Gender, Female, n(%) | 7363(28.59) | 7412(28.79) | 7616(29.58) |
| BMI (kg/m^2^, mean (SD)) | 24.78(1.77) | 26.77(1.90) | 29.31(3.51) |
| WC (cm, mean (SD)) | 88.7(6.8) | 93.2(6.9) | 99.0(8.4) |
| SBP (mmHg, mean (SD)) | 127(18) | 130(17) | 133(17) |
| DBP (mmHg, mean (SD)) | 81(11) | 83(12) | 86(12) |
| Self-reported smoking, n(%) | 3730(14.48) | 3948(15.33) | 3943(15.31) |
| Self-reported drinking, n(%) | 5899(22.91) | 5948(23.10) | 5403(20.98) |
| **Laboratory Examination** | | | |
| FBG (mmol/L, mean (SD)) | 5.65(1.24) | 6.02(1.61) | 6.44(2.00) |
| TC (mmol/L, mean (SD)) | 4.86(0.96) | 4.87(0.97) | 4.91(0.99) |
| TG (mmol/L, mean (SD)) | 2.00(1.64) | 2.26(1.68) | 2.48(1.95) |
| HDL-c (mmol/L, mean (SD)) | 1.23(0.30) | 1.16(0.27) | 1.10(0.26) |
| LDL-c (mmol/L, mean (SD)) | 3.08(0.83) | 3.09(0.84) | 3.10(0.86) |
| ALT (IU/L, mean (SD)) | 19.67(10.39) | 27.36(14.95) | 41.50(27.10) |
| AST (IU/L, mean (SD)) | 20.94(11.13) | 22.13(10.45) | 25.22(12.91) |
| BUN (mmol/L, mean (SD)) | 5.06(1.30) | 5.12(1.24) | 5.18(1.29) |
| Creatinine (μmol/L, mean (SD)) | 71.47(16.39) | 71.50(14.81) | 71.58(17.53) |
| Uric acid (μmol/L, mean (SD)) | 352.91(84.93) | 366.89(84.46) | 380.20(87.11) |
| LEU (×10^9^/L, mean (SD)) | 6.15(1.58) | 6.35(1.65) | 6.71(1.64) |
| RBC (×10^12^/L, mean (SD)) | 4.80(0.44) | 4.90(0.43) | 5.01(0.43) |
| HGB (g/L, mean (SD)) | 147.74(14.36) | 150.84(13.64) | 153.90(13.09) |
| PLT (×10^9^/L, mean (SD)) | 222.46(53.78) | 221.71(53.48) | 222.40(53.52) |
| **Comorbidities** | | | |
| Type 2 diabetes, n(%) | 2737(10.63) | 5387(20.92) | 8353(32.44) |
| Hypertension, n(%) | 9348(36.30) | 11141(43.27) | 13122(50.96) |
| MetS, n(%) | 14988(58.22) | 19870(77.17) | 22417(87.07) |
| Dyslipidaemia, n(%) | 12513(48.59) | 14785(57.42) | 16995(66.00) |
| Hyperuricaemia, n(%) | 3766(14.68) | 4622(18.01) | 5774(22.49) |
| CHD, n(%) | 519(2.02) | 631(2.45) | 628(2.44) |
| Cancer, n(%) | 167(0.65) | 121(0.47) | 99(0.38) |
| Stroke, n(%) | 272(1.06) | 268(1.04) | 262(1.02) |
| CKD, n(%) | 235(0.92) | 245(0.96) | 221(0.86) |

Abbreviations: MAFLD, metabolic dysfunction-associated fatty liver disease; SD, standard deviation; BMI, body mass index; WC, waist circumference; SBP, systolic blood pressure; DBP, diastolic blood pressure; FBG, fasting blood glucose; TC, total cholesterol; TG, triglycerides; LDL-C, low-density lipoprotein cholesterol; HDL-C, high-density lipoprotein cholesterol; ALT, alanine aminotransferase; AST, aspartate transaminase; BUN, blood urea nitrogen; LEU, leukocyte count; RBC, red blood cell; HGB, haemoglobin; PLT, platelet count; MetS, metabolic syndrome; CHD, coronary heart disease; CKD, chronic kidney disease.

**Table S2 Association between nonFLD/FLD-nonMAFLD/MAFLD and Subclinical Carotid Atherosclerosis in the Cross-sectional Analysis**

| **Groups** | **SCA events,** | **Odds ratio (95% confidence interval)** | | | | | |
| --- | --- | --- | --- | --- | --- | --- | --- |
|  | **n/N(%)** | **Crude** | ***p value^c^*** | **Model 1^a^** | ***p value^c^*** | **Model 2^b^** | ***p value^c^*** |
| non-FLD | 26088/69986 (37.28) | Ref | - | Ref | - | Ref | - |
| FLD-nonMAFLD | 803/2232 (35.98) | 0.95 (0.87,1.03) | 0.211 | 1.10 (1.00,1.21) | 0.054 | 1.12 (1.02,1.24) | 0.021 |
| MAFLD | 39029/77251 (50.52) | 1.72 (1.68,1.75) | <0.001 | 1.75 (1.71,1.80) | <0.001 | 1.66 (1.62,1.71) | <0.001 |

Abbreviations: MAFLD, metabolic dysfunction-associated fatty liver disease; FLD, fatty liver disease; SCA,Subclinical Carotid Atherosclerosis.

^a^ Model 1 the adjustment factors included age and sex.

^b^ Model 2 the adjustment factors included age, sex, self-reported smoking, self-reported drinking, red blood cell, leukocyte count, haemoglobin, platelet count, CAD, cancer, stroke and CKD.

^c^ P values were calculated based on Logistic regression.

**Table S3 Association between MAFLD and elevated Carotid Intima-media Thickness/Carotid Plaque in the Cross-sectional Analysis**

| **Groups** | **Outcome** | **Outcome events,** | **Odds ratio (95% confidence interval)** | | | | | |
| --- | --- | --- | --- | --- | --- | --- | --- | --- |
|  |  | **n/N(%)** | **Crude** | p value***^c^*** | **Model 1^a^** | p value***^c^*** | **Model 2^b^** | p value***^c^*** |
| non-MAFLD | Elevated CIMT | 10443/72218 (14.46) | Ref | - | Ref | - | Ref | - |
| MAFLD |  | 18428/77251 (23.85) | 1.85 (1.80,1.90) | <0.001 | 1.76 (1.71,1.81) | <0.001 | 2.08 (2.02,2.14) | <0.001 |
| non-MAFLD | Carotid plaque | 21943/72218 (30.38) | Ref | - | Ref | - | Ref | - |
| MAFLD |  | 31691/77251 (41.02) | 1.59 (1.56,1.63) | <0.001 | 1.66 (1.62,1.71) | <0.001 | 1.53 (1.49,1.57) | <0.001 |

Abbreviations: MAFLD, metabolic dysfunction-associated fatty liver disease.

^a^ Model 1 the adjustment factors included age and sex.

^b^ Model 2 the adjustment factors included age, sex, self-reported smoking, self-reported drinking, red blood cell, leukocyte count, haemoglobin, platelet count, CAD, cancer, stroke and CKD.

^c^ P values were calculated based on Logistic regression.

**Table S4 Baseline Characteristics for the MAFLD HSI Subgroups in the Longitudinal Cohort**

|  | **MAFLD-lowHSI (HSI<35.63)** | **MAFLD-middleHSI (HSI:35.63-39.66)** | **MAFLD-highHSI (HSI>39.66)** |
| --- | --- | --- | --- |
|  | **N=464** | **N=438** | **N=428** |
| Age (year, mean (SD)) | 48.36(6.15) | 47.47(6.50) | 45.68(6.91) |
| Gender, Female, n(%) | 99(21.34) | 72(16.44) | 55(12.85) |
| BMI (kg/m^2^, mean (SD)) | 24.84(1.65) | 26.71(1.71) | 29.34(2.85) |
| WC (cm, mean (SD)) | 88.6(6.3) | 93.1(6.7) | 99.2(8.0) |
| SBP (mmHg, mean (SD)) | 117(16) | 120(15) | 126(16) |
| DBP (mmHg, mean (SD)) | 77(10) | 79(11) | 82(11) |
| Self-reported smoking, n(%) | 12(2.59) | 10(2.28) | 12(2.80) |
| Self-reported drinking, n(%) | 34(7.33) | 16(3.65) | 19(4.44) |
| **Laboratory Examination** | | | |
| FBG (mmol/L, mean (SD)) | 5.42(0.76) | 5.70(1.27) | 6.10(1.72) |
| TC (mmol/L, mean (SD)) | 4.75(0.90) | 4.77(0.85) | 4.85(0.99) |
| TG (mmol/L, mean (SD)) | 1.95(1.64) | 2.31(2.02) | 2.56(2.29) |
| HDL-c (mmol/L, mean (SD)) | 1.20(0.29) | 1.08(0.24) | 1.03(0.26) |
| LDL-c (mmol/L, mean (SD)) | 3.06(0.77) | 3.10(0.75) | 3.10(0.82) |
| ALT (IU/L, mean (SD)) | 20.21(9.58) | 28.05(12.34) | 42.80(23.60) |
| AST (IU/L, mean (SD)) | 20.05(7.97) | 21.58(8.54) | 24.78(10.72) |
| BUN (mmol/L, mean (SD)) | 5.05(1.22) | 5.06(1.13) | 5.13(1.10) |
| Creatinine (μmol/L, mean (SD)) | 70.91(14.10) | 70.97(14.35) | 69.46(12.45) |
| Uric acid (μmol/L, mean (SD)) | 363.20(83.04) | 378.04(83.14) | 390.03(84.03) |
| LEU (×10^9^/L, mean (SD)) | 5.80(1.31) | 5.98(1.38) | 6.33(1.46) |
| RBC (×10^12^/L, mean (SD)) | 4.78(0.44) | 4.91(0.41) | 4.98(0.41) |
| HGB (g/L, mean (SD)) | 147.25(14.60) | 150.64(12.49) | 152.49(12.59) |
| PLT (×10^9^/L, mean (SD)) | 219.30(53.89) | 217.55(49.33) | 222.04(51.16) |
| **Comorbidities** | | | |
| Type 2 diabetes, n(%) | 25(5.39) | 49(11.19) | 114(26.64) |
| Hypertension, n(%) | 68(14.66) | 86(19.63) | 125(29.21) |
| MetS, n(%) | 208(44.92) | 295(67.35) | 338(78.97) |
| Dyslipidaemia, n(%) | 206(44.40) | 247(56.39) | 292(68.22) |
| Hyperuricaemia, n(%) | 75(16.16) | 91(20.82) | 111(26.00) |
| CHD, n(%) | 0(0) | 0(0) | 0(0) |
| Cancer, n(%) | 0(0) | 0(0) | 0(0) |
| Stroke, n(%) | 0(0) | 0(0) | 0(0) |
| CKD, n(%) | 1(0.22) | 1(0.23) | 1(0.23) |

Abbreviations: MAFLD, metabolic dysfunction-associated fatty liver disease; SD, standard deviation; BMI, body mass index; WC, waist circumference; SBP, systolic blood pressure; DBP, diastolic blood pressure; FBG, fasting blood glucose; TC, total cholesterol; TG, triglycerides; LDL-C, low-density lipoprotein cholesterol; HDL-C, high-density lipoprotein cholesterol; ALT, alanine aminotransferase; AST, aspartate transaminase; BUN, blood urea nitrogen; LEU, leukocyte count; RBC, red blood cell; HGB, haemoglobin; PLT, platelet count; MetS, metabolic syndrome; CHD, coronary heart disease; CKD, chronic kidney disease.

**Table S5 Association between nonFLD/FLD-nonMAFLD/MAFLD and Subclinical Carotid Atherosclerosis in the Longitudinal Cohort**

| **Groups** | **SCA events,** | **Hazards ratio (95% confidence interval)** | | | | | |
| --- | --- | --- | --- | --- | --- | --- | --- |
|  | **n/N(%)** | **Crude** | ***p value^c^*** | **Model 1^a^** | ***p value^c^*** | **Model 2^b^** | ***p value^c^*** |
| non-FLD | 231/808 (28.59) | Ref | - | Ref | - | Ref | - |
| FLD-nonMAFLD | 12/41 (29.27) | 1.22 (0.68,2.18) | 0.503 | 1.28 (0.71,2.28) | 0.412 | 1.35 (0.75,2.42) | 0.312 |
| MAFLD | 642/1330 (48.27) | 1.52 (1.31,1.77) | <0.001 | 1.32 (1.12,1.55) | <0.001 | 1.33 (1.13,1.57) | <0.001 |

Abbreviations: MAFLD, metabolic dysfunction-associated fatty liver disease; FLD, fatty liver disease; SCA,Subclinical Carotid Atherosclerosis.

^a^ Model 1 the adjustment factors included age and sex.

^b^ Model 2 the adjustment factors included age, sex, self-reported smoking, self-reported drinking, red blood cell, leukocyte count, haemoglobin, platelet count and CKD.

^c^ P values were calculated based on Cox regression.

**Table S6 Association between MAFLD and elevated Carotid Intima-media Thickness/Carotid Plaque in the Longitudinal Cohort**

| **Groups** | **Outcome** | **Outcome events,** | **Hazards ratio (95% confidence interval)** | | | | | |
| --- | --- | --- | --- | --- | --- | --- | --- | --- |
|  |  | **n/N(%)** | **Crude** | ***p value^c^*** | **Model 1^a^** | ***p value^c^*** | **Model 2^b^** | ***p value^c^*** |
| non-MAFLD | Elevated CIMT | 185/849  (21.79) | Ref | - | Ref | - | Ref | - |
| MAFLD |  | 517/1330  (38.87) | 1.60 (1.35,1.89) | <0.001 | 1.38 (1.15,1.65) | <0.001 | 1.38 (1.15,1.66) | <0.001 |
| non-MAFLD | Carotid plaque | 113/849  (13.31) | Ref | - | Ref | - | Ref | - |
| MAFLD |  | 310/1330  (23.31) | 1.57 (1.26,1.94) | <0.001 | 1.28 (1.02,1.61) | 0.037 | 1.31 (1.04,1.65) | 0.024 |

Abbreviations: MAFLD, metabolic dysfunction-associated fatty liver disease; CIMT, carotid intima-media thickness.

^a^ Model 1 the adjustment factors included age and sex.

^b^ Model 2 the adjustment factors included age, sex, self-reported smoking, self-reported drinking, red blood cell, leukocyte count, haemoglobin, platelet count and CKD.

^c^ P values were calculated based on Cox regression.

**Table S7 Sensitivity Test I for the Incidence of Subclinical Carotid Atherosclerosis in MAFLD group versus non-MAFLD group during Follow-up Time above 2 Years**

| **Groups** | SCA events,  **n/N(%)** | **Hazards ratio (95% confidence interval)** | | | | | |
| --- | --- | --- | --- | --- | --- | --- | --- |
|  |  | **Crude** | p valuec | Model 1a | p valuec | Model 2b | p valuec |
| **non-MAFLD vs. MAFLD** | | | | | | | |
| non-MAFLD | 142/459(30.94) | Ref | - | Ref | - | Ref | - |
| MAFLD | 391/771(50.71) | 1.49(1.23,1.81) | <0.001 | 1.34(1.08,1.65) | 0.007 | 1.37(1.11,1.70) | 0.003 |
| **non-MAFLD vs. MAFLD subgroups** | | | | | | | |
| Non-MAFLD | 142/459(30.94) | Ref | - | Ref | - | Ref | - |
| MAFLD-lowHSI(HSI<35.63) | 126/263(47.91) | 1.51(1.19,1.92) | <0.001 | 1.28(0.99,1.65) | 0.055 | 1.32(1.03,1.71) | 0.031 |
| MAFLD-middleHSI(HSI:35.63-39.65) | 121/250(48.40) | 1.50(1.17,1.91) | 0.001 | 1.33(1.02,1.72) | 0.032 | 1.35(1.04,1.75) | 0.026 |
| MAFLD-highHSI(HSI>39.65) | 144/258(55.81) | 1.47(1.16,1.85) | 0.001 | 1.40(1.09,1.79) | 0.008 | 1.46(1.13,1.88) | 0.004 |

Abbreviations: MAFLD, metabolic dysfunction-associated fatty liver disease; SCA,Subclinical Carotid Atherosclerosis.

^a^ Model 1 the adjustment factors included age and sex.

^b^ Model 2 the adjustment factors included age, sex, self-reported smoking, self-reported drinking, red blood cell, leukocyte count, haemoglobin, platelet count and chronic kidney disease.

^c^ P values were calculated based on Cox regression.

**Table S8 Sensitivity Test II for the Incidence of Subclinical Carotid Atherosclerosis in MAFLD Group versus non-MAFLD Group after further adjusting FIB-4**

| **Groups** | **Hazards ratio (95% confidence interval)** | |
| --- | --- | --- |
|  | **Model 3**a | ***p value^b^*** |
| **non-MAFLD vs. MAFLD** | | |
| non-MAFLD | Ref | - |
| MAFLD | 1.31(1.11,1.54) | 0.001 |
| **non-MAFLD vs. MAFLD subgroups** | | |
| Non-MAFLD | Ref | - |
| MAFLD-lowHSI(HSI<35.63) | 1.23(1.02,1.50) | 0.034 |
| MAFLD-middleHSI(HSI:35.63-39.65) | 1.33(1.10,1.63) | 0.004 |
| MAFLD-highHSI(HSI>39.65) | 1.37(1.13,1.67) | 0.002 |

Abbreviations: MAFLD, metabolic dysfunction-associated fatty liver disease.

^a^ Model 3 the adjustment factors included age, sex, self-reported smoking, self-reported drinking, red blood cell, leukocyte count, haemoglobin, platelet count, chronic kidney disease and FIB-4.

^b^ P values were calculated based on Cox regression.

**Table S9 Sensitivity Test III for the incidence of Subclinical Carotid Atherosclerosis in MAFLD Group versus Non-MAFLD Group after further adjusting Dyslipidemia**

| **Groups** | **Hazards ratio (95% confidence interval)** | |
| --- | --- | --- |
|  | **Model 3 ^a^** | ***p value^b^*** |
| **non-MAFLD vs. MAFLD** | | |
| non-MAFLD | Ref | - |
| MAFLD | 1.29(1.10,1.53) | 0.002 |
| **non-MAFLD vs. MAFLD subgroups** | | |
| Non-MAFLD | Ref | - |
| MAFLD-lowHSI(HSI<35.63) | 1.23(1.01,1.49) | 0.042 |
| MAFLD-middleHSI(HSI:35.63-39.65) | 1.32(1.08,1.62) | 0.006 |
| MAFLD-high-HSI(HSI>39.65) | 1.36(1.11,1.66) | 0.003 |

Abbreviations: MAFLD, metabolic dysfunction-associated fatty liver disease.

^a^ Model 3 the adjustment factors included age, sex, self-reported smoking, self-reported drinking, red blood cell, leukocyte count, haemoglobin, platelet count, chronic kidney disease and dyslipidemia.

^b^ P values were calculated based on Cox regression.
